# Knowledge, attitudes, and practices toward rabies in three provinces of Indonesia

**DOI:** 10.14202/vetworld.2021.2518-2526

**Published:** 2021-09-25

**Authors:** Saifur Rehman, Fedik Abdul Rantam, Abdul Rehman, Mustofa Helmi Effendi, Aamir Shehzad

**Affiliations:** 1 Division of Veterinary Public Health, Faculty of Veterinary Medicine, Universitas, Airlangga, Surabaya Indonesia; 2 Department of Epidemiology and Public Health, University of Veterinary and Animal Sciences, Lahore, Pakistan; 3 Laboratory of Virology and Immunology, Division of Microbiology, Faculty of Veterinary Medicine, Universitas Airlangga, Surabaya, Indonesia.

**Keywords:** awareness, Bali, East Java, Indonesia, rabies, West Nusa Tenggara, zoonotic disease

## Abstract

**Background and Aim:**

Rabies is an important viral zoonotic disease that is mostly transmitted through the bite of a rabid dog. Despite serious efforts regarding its control, rabies is still endemic in many provinces of Indonesia. The study aims to assess the knowledge, attitudes, and practices (KAP) related to rabies in urban and rural areas in three provinces of Indonesia.

**Materials and Method:**

s: A total of 432 respondents of different age groups, educational levels, geographical areas, and occupations participated in this study. Data were collected using a pre-designed questionnaire with online and offline modes to assess the KAP of rabies among the respondents. A series of Chi-square tests and frequency distribution analyses were performed to determine associations between response variables.

**Results:**

Of the 432 participants, 56.9% were aware of the clinical signs of rabies. Excepting for people at high risk of contracting the disease (e.g., veterinarians), most respondents (83.1%) were not vaccinated against rabies. Surprisingly, 79.4 % of those who were bitten by an infected dog did not seek medical care from the doctor and approximately 71.8% had poor knowledge of rabies control and vaccine campaigns. Of all respondents, 64% (p<0.05, odds ratio=1.63) were vaccinated after an infected dog bite. Similarly, 32% (p<0.05, odds ratio=1.59) were aware of surveys and vaccinations in their areas. In contrast, 20.7% (p<0.05, odds ratio=0.593) reported that rabid dogs were killed in their areas. The majority (89.60%) of the respondents were aware of the fact that rabies can cause death. Most of the respondents (93%) knew that rabies is caused by an infected dog bite. The overall levels of KAP among the respondents were good.

**Conclusion:**

The findings of the current study generally show that participants had good knowledge about clinical signs based on their frequency percentage, but lacked knowledge regarding medical treatment and surveys for awareness and vaccination of rabies. Overall, a significant (p<0.05, odds ratio>1) relationship was found among the KAP of participants. This depicts that the majority of the population is aware of rabies and factors involved in its transmission.

## Introduction

Rabies is one of the most important and dangerous viral zoonosis caused by rabies virus, which belongs to the genus lyssavirus and family Rhabdoviridae. It kills over 60,000 people annually worldwide. The disease mainly affects children younger than 10 years old in Africa and Asia [1]. However, the true number of deaths is thought to be as high as 100,000 annually [2]. It is estimated that for every case reported, as many as 10 cases may go unreported [3]. According to the World Health Organization (WHO) and Center for Disease Control, all continents of the world except Antarctica have been affected by rabies with more than 95% of deaths occurring in Africa and Asia [4]. Domestic dogs are the main transmitters of human rabies, which can be prevented using personal protective equipment (PPE); however, same is not readily available in underdeveloped countries [5]. Control and elimination of human rabies primarily depends on controlling rabies in the dog population. Southeast Asian countries can be categorized depending on rabies status: Low, medium, high, and rabies-free countries. Because of the increased incidence of rabies, Indonesia is moving from a low to medium rabies endemic country. Rabies is an emerging disease problem in many islands of Indonesia which were previously considered rabies free. Because of competing public health priorities and the complex nature of rabies control activities, the disease is still neglected in many countries [6]. Although rabies is preventable, a lack of education and the high cost of vaccines limit the use of PPE. Furthermore, a lack of awareness about the disease causes it to remain endemic. A previous study shows that most persons become affected with rabies secondary to ignorance, negligence, and lack of primary health-care services [7].

Knowledge, attitude, and practice (KAP) surveys are extensively used worldwide for public health-related research primarily based on the precept that expertise and knowledge will increase the health-related attitudes and practices of decreasing the chances of the disease [8]. A recent KAP survey was conducted in Thailand to increase the awareness in the community regarding controlling and preventing dengue [9]. Similarly, KAP surveys recognize the behavior, culture confidence, and gaps which might also pose as boundaries to controlling and preventing infectious zoonotic diseases [10].

KAP surveys have been implemented for studying rabies and generating baseline records that are essential for creating knowledge, awareness, and practices to control and prevent rabies [11]. Rabies was first reported in Indonesia in 1884 and is now considered to be endemic in more than 20 of its provinces [12]. According to data from the Indonesian Ministry of Health, an estimated 100,000 dog bites occur each year, resulting in 150-300 cases of human rabies [13]. The Indonesian rabies virus lineage remains closely related to the Asian lineage, lyssavirus genotype 1 [14]. Many pilot studies and control programs are regularly implemented at the district and provincial levels in Indonesia, but because of the lack of vaccination coverage in humans and animals, the disease is still endemic in the country. Rabies was first reported in Bali islands in 2008 and is thought to have been introduced by fishermen from the island of Sulawesi (Indonesia). Many subsequent pilot studies and vaccination campaigns were functional on the island; however, the disease remains endemic in the island, with most cases originating from the island of Sulawesi [15]. There were more than 500,000 cases of animal rabies reported in Indonesia between 2011 and 2017. As many as, 836 cases of human rabies were reported on August 27, 2018 in Jakarta [16]. Of the 34 provinces of Indonesia, only nine are declared rabies free, but the disease is still endemic in many parts of the country. In our findings, most of the respondents were vaccinating their pets, which are an important step in controlling rabies as compared to previous KAP surveys in Pakistan, Ethiopia, Grenada, and India [17-19] where most pets are unvaccinated. Vaccination is an important factor for preventing disease transmission from pets, especially dogs, to humans.

Our findings are expected to guide decision makers to improve rabies prevention and control in dogs and in humans through targeted community-based education programs regarding KAP of rabies.

The study aims to determine the level of KAP regarding rabies in the general population of three provinces of Indonesia.

## Materials and Methods

### Ethical approval and Informed consent

This study was approved by the Animal Care and Use Committee, Faculty of the Veterinary Medicine, University of Airlangga, Surabaya by approval letter No: 1.KE.012.02.2021. The informed consent was obtained from all the participants before the study.

### Study period and area

**Figure-1:**
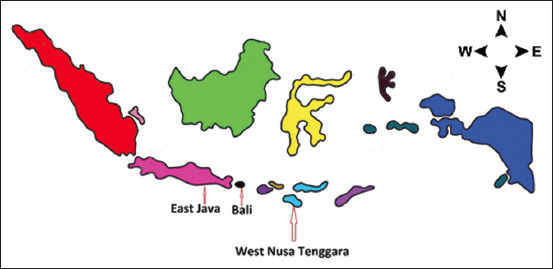
Indonesian map showing the sampling areas [Source: humdata.org].

**Figure-2:**
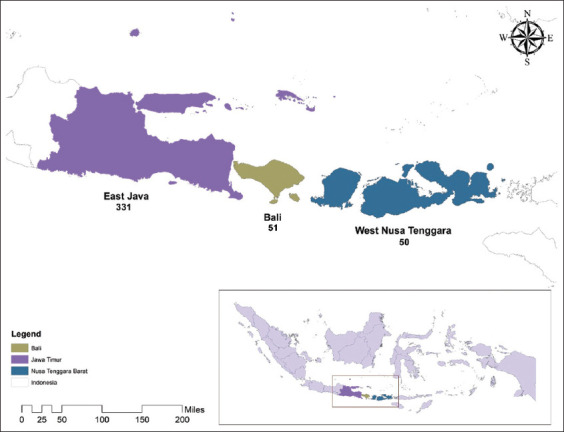
Study area [Source: ArcGIS version 10.7].

This KAP survey was conducted from September 2020 to January 2021. During this period, data were collected from the general population among different regions of three provinces of Indonesia. A community-based cross-sectional survey involving online and face-to-face interviews was conducted in East Java, Bali, and West Nusa Tenggara provinces of Indonesia. East Java is Indonesia’s rugged province, which includes Madura, some of the coastal towns, and the eastern part of Java. The total area of East Java is 47,800 km^2^ and the total population is 38.85 million. Surabaya is the capital city located within the northern coastal part of the province of East Java within the south-central region of Indonesia and an important hub of social lifestyle. Bali is one of the reputed luxury accommodations and a beautiful tourist region in the far east of Indonesia. Bali is positioned to the east of the island of Java and is connected with the large island through a common ferry line. West Nusa Tenggara is one of the provinces in central Indonesia that is situated on the Less Sunda islands (Figures-1 and 2). The population of the province is approximately 4.7 million people. The province consists of eight regencies and two municipalities [20]. Our KAP survey covered approximately 5.3% (100,826/1,904,569 km^2^) of the country’s landmass.

### Sampling techniques and sample size

Online and offline cross-sectional surveys were conducted among the general population of three provinces of Indonesia. Online data were collected through Google Forms by creating a link that was shared through WhatsApp and emails to approximately 600 people of three provinces. Offline data were collected through face-to-face interviews with the consent of respondents. The questionnaire was originally designed in the English language; however, it was translated into the local language of that region to increase the accuracy of the respondent rates, decrease the margin of errors, and avoid confusion among the respondents. A total of 432 respondents, comprising 202 males and 230 females from different cities and villages of three provinces (East Java, West Nusa Tenggara, and Bali) of Indonesia, completed the questionnaire. The online questionnaire was forwarded to 600 participants, of which 245 accessed the form (41% response rate). Thirteen responses were incomplete and were removed from the final analysis. Two hundred questionnaires were collected through face-to-face interviews. The entire data were collected with the help of veterinary doctors, university friends, and other local people working in different government and private sectors. They shared the questionnaire with friends through WhatsApp and emails. In Bali and West Nusa Tenggara provinces, we shared the questionnaires through WhatsApp and emails because these provinces were far away from us, but in East Java, we completed the questionnaires through WhatsApp, emails, and face-to-face interviews. The targeted population was the general public aged <20 years, 20-30 years, and 31-50 years. A brief description about the study was given to respondents before data collection.

### Questionnaire survey

The questionnaire was divided into three sections. Section one contained six questions regarding the demographic variables of the respondents. The second section consisted of 11 questions related to the awareness and knowledge of the respondents about rabies, while the third section had six questions on practices and attitudes of rabies among respondents. The questionnaire was designed to follow the KAP surveys on rabies that was earlier conducted in many parts of the world. To include respondents from diverse geographical backgrounds, the questionnaire was sent to urban and rural communities in the provinces. The first part of the study questionnaire collected the demographical data of the respondents that represented the different geographical areas based on age, occupation, education, and gender. The second and third parts of the study questionnaire were designed in such a way to evaluate the knowledge and awareness related to the clinical signs and symptoms, transmission, and zoonotic importance of rabies as mentioned in already published literature [21].

Acknowledgment of rabies prevention and control was estimated about respondent’s attitudes and practices related to pre-exposure and post-exposure prophylaxis. Moreover, KAP of respondents regarding prevention and control of rabies was estimated through self and pet vaccination. The knowledge and awareness to recognize rabid animals were also included in the questionnaire in relation to rabies control and prevention strategies.

### Study questionnaire

Questionnaire and supplementary data can be available from the corresponding author upon a reasonable request.

### Statistical analysis

Data were collected on a structured questionnaire with the help of veterinarians. The participant’s KAP regarding rabies was assessed through the structured questionnaire. The data were entered into a Microsoft® Excel (Microsoft Corporation, Washington, USA) spreadsheet and statistical analysis was performed using Statistical Package for the Social Sciences version 25.0(IBM, NY, USA). Initially, descriptive analysis and univariate analysis were performed to estimate respondent’s KAP related to rabies in the form of percentages. The association was considered significant if p<0.05. A frequency table was used to display the awareness, knowledge, and level of education of the participant in terms of absolute numbers and percentages. We divided the respondents on the basis of their education level (non-formal, primary, secondary, and tertiary) to assess and compare each respondent’s baseline knowledge and awareness of rabies. Each respondent’s level of education and their awareness were displayed using a frequency table in absolute numbers and percentages. Outcomes from the final models have been expressed in phrases of odds ratios related to 95% confidence intervals. Cross-tabulation and Chi-square analysis were performed to assess the association between outcome and test variables (Table-1). We divided the levels of those with good/poor knowledge, good/bad attitudes, and appropriate/inappropriate practices based on their percentage.

**Table-1:** Detailed analysis of participant responses about KAP survey of rabies.

Questionnaire	Participant response	Participant n=432		OR	Confidence interval	p-value

		Male	Female			
Do you know rabies causes through dog bites?	Yes	188	224	2.780	1.048-7.377	0.033
	No	14	6			
Do you know rabies can cause death?	Yes	181	221	2.849	1.273-6.374	0.008
	No	21	9			
Do you know the role of dogs in the transmission of rabies?	Yes	176	210	1.551	0.837-2.873	1.60
	No	26	20			
Do you know about the vaccination of rabies?	Yes	151	187	1.469	0.928-2.324	0.10
	No	51	43			
Do you know about taking vaccination of rabies after dog biting?	Yes	107	149	1.633	1.109-2.404	0.013
	No	95	81			
Do you know that vaccination of rabies is better before dog biting?	Yes	104	138	1.413	0.965-2.070	0.075
	No	98	92			
Any survey held about awareness and vaccination of rabies in your area?	Yes	47	75	1.596	1.041-2.446	0.031
	No	155	155			
Do you know any patient victims of rabies in your family or area?	Yes	32	30	0.797	0.465-1.365	0.408
	No	170	200			
Do you know about the clinical signs of rabies?	Yes	109	137	1.257	0.858-1.842	0.240
	No	93	93			
Do you know after a rabid dog bite any doctor visited in your area?	Yes	42	47	0.978	0.613-1.561	0.927
	No	160	183			
Are there any pets or other animals in your house?	Yes	144	176	1.313	0.853-2.020	0.215
	No	58	54			
Are you usually vaccinating your pets?	Yes	105	128	1.159	0.793-1.694	0.445
	No	97	102			
Are you vaccinated against rabies?	Yes	28	45	1.512	0.903-2.530	0.114
	No	174	185			
Are you killing rabid dogs in your area?	Yes	42	31	0.593	0.357-0.987	0.043
	No	160	199			
Do you consider rabies vaccination is beneficial for humans?	Yes	180	212	1.440	0.749-2.768	0.273
	No	22	18			
Do you know rabies is a zoonotic disease?	Yes	177	210	1.483	0.797-2.760	0.221
	No	25	20			
Do you use any medication at the home after a dog bite?	Yes	106	103	0.735	0.503-1.073	0.110
	No	96	127			

OR=Odds ratio

## Results

We interviewed 432 respondents from different geographical regions of Indonesia. We selected three provinces (East Java, Bali, and West Nusa Tenggara) in our KAP survey. Of the 432 respondents, 331 (76.6%) were from East Java, 51 (11.8%) from Bali, and 50 (11.6%) from West Nusa Tenggara. Among the 432 respondents, 222 (51.4%) lived in urban areas, while 210 (48.6%) belonged to rural areas. Of the overall respondents, female respondents represented 53.2%, while male respondents accounted for 46.8% of the collected data. In this survey, the data were collected from a general population aged <20 years (17.1%), between 20 and 30 years (66%), and between 31 and 50 years (16.9%). Of all the respondents, 71.3% had completed or were undertaking their tertiary (bachelors and masters) education, and 25.4% had completed their secondary (matric and intermediate) education (Table-2).

**Table-2:** Descriptive statistics of demographic variables of respondents (n=432).

Type of variables	Participant response	Frequency	Percentage
Gender	Male	202	46.8
	Female	230	53.2
Age	<20 years	74	17.1
	20-30 years	285	66.0
	31-50 years	73	16.9
Qualification	Non-formal (illiterate)	8	1.9
	Primary (middle)	6	1.4
	Secondary (matric, intermediate)	110	25.4
	Tertiary (bachelor, master)	308	71.3
Occupation	Unemployed	191	44.21
	Businessman	74	17.13
	Farmworker	58	13.43
	Professional	109	25.23
Geographical distribution	Urban	222	51.4
	Rural	210	48.6
Study area	Bali	51	11.8
	East Java	331	76.6
	West Nusa Tenggara	50	11.6

Table-3 shows that 412 (95.4%) respondents were aware that rabies is caused by infected dog bites, while 402 (93%) knew that rabies can cause death. The majority of the respondents (386 [89.4%]) were aware that dogs have an important role in rabies transmission. Pertaining to knowledge and awareness about the vaccination of rabies, 338 (78.2%) respondents had knowledge about vaccination, while 256 (59.4%) believed that vaccination is best if given before dog bite. In the current study, the majority (310 [71.8%]) of the respondents did not know about the previous survey held in their area. We found that only 62 (14.4%) respondents knew about victims of rabies, or patients in their families or areas. Overall, 343 (79.4%) respondents said that no doctor visited their area after a rabid dog bite. In this study, most of the respondents (392 [90.1%]) knew that rabies is preventable through vaccination. Regarding the zoonotic importance of rabies, 392 (90.7%) respondents knew that rabies is a fatal zoonotic disease (Table-3).

**Table-3:** Descriptive statistics of knowledge and awareness of rabies among the respondents (n = 432).

Name of variables	Participant response	Frequency	Percentage
Do you know rabies is caused by dog bites?	Yes	412	95.4
	No	20	4.6
Do you know rabies can cause death?	Yes	402	93.1
	No	30	6.9
Do you know the role of dogs in the transmission of rabies?	Yes	386	89.4
	No	46	10.6
Do you know about the vaccination of rabies?	Yes	338	78.2
	No	94	21.8
Do you know about taking vaccination of rabies after dog biting?	Yes	256	59.3
	No	176	40.7
Do you know that vaccination of rabies is better before dog biting?	Yes	242	56.0
	No	190	44.0
Any survey held about awareness and vaccination of rabies in your area?	Yes	122	28.2
	No	310	71.8
Do you know any patient victims of rabies in your family or area?	Yes	62	14.4
	No	370	85.6
Do you know about the clinical signs of rabies?	Yes	246	56.9
	No	186	43.1
Do you know after the rabid dog bite any doctor visited in your area?	Yes	89	20.6
	No	343	79.4
Do you know rabies is a zoonotic disease?	Yes	387	89.6
	No	45	10.4

Table-4 reveals that 320 (74.1%) respondents said that they had pets and other animals in their houses, and more than half (233 [53.9%]) of them vaccinated their pets. Only 73 (16.9%) respondents answered that they were vaccinated against rabies and killed rabid dogs with the assistance of a relevant authority and by following the proper protocols issued by the state. Home medication after an infected dog bite was practiced by 209 (48.4%) respondents. Table-1 shows the detailed analysis of the respondent’s KAP. Of the overall responses, only five showed a significant association with outcomes based on p-value. The current study reveals that rabies can cause death, and that vaccination after dog bites, awareness of vaccination in your area, and killing rabid dogs in your area showed a significant association with study outcomes (Table-1). Table-5 shows the relationships of education level with rabies awareness, clinical signs of a rabid animal, and the practice of seeking medical assistance after suspected animal bite in percentages. No respondent answered yes.

**Table-4:** Descriptive statistics of respondents’ attitude and practices regarding rabies.

Name of variables	Participant response	Frequency	Percentage
Are there any pets or other animals in your house?	Yes	320	74.1
	No	112	25.9
Are you usually vaccinating your pets?	Yes	233	53.9
	No	199	46.1
Are you vaccinated against rabies?	Yes	73	16.9
	No	359	83.1
Are you killing rabid dogs in your area?	Yes	73	16.9
	No	359	83.1
Do you consider rabies vaccination is beneficial for humans?	Yes	392	90.7
	No	40	09.3
Do you use any medication in the home after a dog bite?	Yes	209	48.4
	No	223	51.6

**Table-5:** Cross-tabulation of respondents’ qualification with rabies awareness to clinical signs, surveys, and practices of seeking doctor after a dog bite.

Qualification	Non-formal (illiterate)	Primary (middle)	Secondary (matric + intermediate)	Tertiary (bachelor + master)
Awareness to clinical signs	12.5	**	37.27	66.23
Awareness about rabies survey and vaccination	12.5	33.33	23.63	30.19
Doctor visits after infected dog bite	25	16.67	19.09	21.10

** No respondents answer yes

In Bali and West Nusa Tenggara Provinces, we shared the questionnaires through WhatsApp and emails because these provinces were far away from us; however, in East Java, we completed the questionnaires through WhatsApp, emails, and through face-to-face interviews. The questionnaire was divided into three sections. Section one contained six questions regarding the demographic variables of the respondents. The second section consisted of 11 questions related to the awareness and knowledge of the respondents about rabies, while the third section had six questions on practices and attitudes of rabies among respondents. Table-6 shows the predictable variables correlation with KAP of rabies among the respondents.

**Table-6:** Predictable variables correlation with KAP of rabies among the respondents.

KAP of rabies	Gender	Age	Qualification	Occupation
Do you know rabies causes through dog bites?				
R	0.103*	0.094	0.277**	0.035
Sig	0.033	0.052	0.000	0.470
Do you know rabies can cause death?				
R	0.127**	−0.064	0.182**	0.087
Sig	0.008	0.188	0.000	0.070
Do you know the role of dogs in transmission of rabies?				
R	0.068	0.050	0.122*	0.061
Sig	0.161	0.299	0.011	0.208
Do you know about the vaccination of rabies?				
R	0.079	0.084	0.222**	0.169**
Sig	0.100	0.079	0.000	0.000
Do you know about taking vaccination of rabies after dog biting?				
R	0.120*	0.182**	0.117*	0.101*
Sig	0.013	0.000	0.015	0.036
Do you know that vaccination of rabies is better before dog bite?				
R	0.086	0.116*	0.174**	0.178**
Sig	0.076	0.015	0.000	0.000
Any survey held about awareness and vaccination of rabies in your area?				
R	0.104*	0.029	0.086	0.232**
Sig	0.031	0.549	0.073	0.000
Do you know any patient victim of rabies in your family or area?				
R	−0.040	0.024	−0.046	0.089
Sig	0.409	0.615	0.343	0.064
Do you know about the clinical signs of rabies?				
R	0.056	0.173**	0.295**	0.224**
Sig	0.241	0.000	0.000	0.000
Do you know after rabid dog bite any doctor visited in your area?				
R	−0.004	0.139**	−0.006	0.113*
Sig	0.927	0.004	0.906	0.019
Are there any pet or other animals in your house?				
R	0.060	−0.075	−0.102*	0.026
Sig	0.216	0.121	0.034	0.595
Are you usually vaccinating your pets?				
R	0.037	−0.067	0.032	0.131**
Sig	0.446	0.162	0.512	0.006
Are you vaccinated against rabies?				
R	0.076	0.002	0.109*	0.182**
Sig	0.115	0.970	0.024	0.000
Are you killing rabid dogs in your area?				
R	−0.097*	0.023	0.067	0.098*
Sig	0.043	0.634	0.163	0.043
Do you consider rabies vaccination is beneficial for humans?				
R	0.053	−0.015	0.073	0
Sig	0.274	0.757	0.131	0.295
Do you know rabies is a zoonotic disease?				
R	0.060	0.116*	0.235**	0.176**
Sig	0.212	0.016	0.000	0.000
Do you use any medication in home after dog bite?				
R	−0.077	0.155**	0.143**	0.208**
Sig	0.111	0.001	0.003	0.000

KAP=Knowledge, attitude, and practice, R= Correlation, Sig= Significant

## Discussion

This study aimed to understand the level of KAP regarding rabies among the general population of three provinces of Indonesia. To our understanding, this is the first KAP study on rabies that covers three provinces of the country. The current study shows that most of the participants had sufficient knowledge and appropriate practices regarding rabies. However, there are some gaps in knowledge and practices among the participants, especially on rabies vaccination, doctors’ visits to the affected areas, and home medications after an infected dog bite. Our findings are expected to guide decision-makers in improving rabies prevention and control in dogs and humans through targeted community-based education programs regarding KAP of rabies.

Rabies remains a major global public health issue, especially in developing countries such as Indonesia, Pakistan, and India. Since rabies is considered a non-communicable disease with limited public knowledge, awareness campaigns are being conducted worldwide. The WHO recently worked under the umbrella of “Zero Rabies by 2030” and caused several countries to start efforts to minimize the risk of rabies from dog bites [22]. In terms of basic local community information, we presumed that the majority of the population may have general knowledge and appropriate understanding of clinical signs, transmission, and preventive measures for controlling rabies associated with infected dog bites. This main hypothesis shall be examined with the help of the following subhypothesis.

The population adopts appropriate practices to prevent any interaction with rabid animals or rabies based on the basic knowledge of rabies disease. Our results demonstrated significant aspects concerning the degree of knowledge of individuals that were known to be at high risk of rabies. Our findings showed that 95.4% of respondents were aware that rabies is caused by infected dog bites. This is a significant factor in terms of the knowledge to control rabies. Similarly, like many other parts of the globe, the majority of respondents in this study had pets or domestic ­animals [21]. In our findings, most of the respondents (233 [53.9%]) were vaccinating their pets, which are an important step regarding controlling rabies, as compared to previous KAP surveys in Pakistan, Ethiopia, Grenada, and India [17-19] in which most persons did not vaccinate their pets [19,23]. We also found that many of our respondents were aware of rabies and its deadly existence, and the clinical signs associated with rabies. These results are different from the past research in Philippines, Bangladesh, and Tanzania [24-26]. One of the critical findings of this research is that 51.6% of respondents did not seek immediate medical attention after an infected dog bite, which was somewhat comparable with similar rabies studies in Pakistan [27], but in contrast to previously published studies on rabies in developed nations across the globe [11,18,21]. Most of our respondents (more than 65%) had strong knowledge regarding the potential source, prevention, and control of rabies in the study area. Approximately 71.8% of respondents were not aware of any vaccine activities (any survey or vaccination campaign related to rabies) or rabies initiatives in the study area. This result is a non-essential attempt to monitor and eradicate rabies in the study area. Rabies awareness in terms of transmission, etiology, major host, and reservoirs are important to reduce the number of rabies cases in Indonesia. In our survey, most of the participants knew that rabies is prevented by vaccination, and most were aware that a dog bite is the main source of rabies transmission. Our results were similar to those from other regional countries [12,28], but most of the participants had little knowledge regarding awareness and vaccination campaigns against rabies in the study area. It was also observed that 48.4% of people seek traditional remedies and spiritual healers to cure rabies instead of visiting hospitals. This practice of seeking a spiritual healer for a possible rabies patient is also reported in Tanzania, Ethiopia, and India [26,29,30]. Although we did not directly investigate this problem throughout the survey, this may also explain why our research revealed that 51.6% of participants would not pursue conventional medical treatment. They may have sought out charismatic herbal remedies, most of which give free treatment. That is why we should feel it reasonable and appropriate to involve traditional herbal remedies in our efforts in Indonesia to eradicate rabies. If we provide resources to them, these charismatic therapists and practitioners can also act as important platforms for individuals and community leaders, and can also help to determine the true impact of rabies in Indonesia [24].

The sampling method and research area covered are our main study limitations since these findings could not be extended to all of Indonesia. The lack of a scoring system is another caveat that makes it hard to fully evaluate the overall frame. Our survey did not have sufficient financial and social resources to cover other provinces of Indonesia, such as West Java, where rabies is still endemic. In March 2020, 15 persons died in West Nusa Tenggara, Dompu Regency, secondary to the lack of vaccination and awareness regarding rabies [31]. Several other regions in Indonesia have insufficient data on rabies-related deaths. This study recommends that additional detailed data on rabies throughout all areas of Indonesia be applied to all areas and regions. The main asset of this study is its validity. This study is the first study in Indonesia to cover three provinces with a sample size of 432. Eventually, this research would lead to future studies aimed at reducing the number of deaths associated with rabies in Indonesia. Rabies is gradually contributing to deaths that create public outrage, mainly because of a miserable death and the unavailability of rabies vaccine. This KAP study also aims to guide the efforts to enhance health-seeking actions, such as prophylaxis after infection and pursuing medical treatment after the dog bite. The outcome of this research will potentially increase awareness, attitudes, and practices of local rabies throughout the study area and eventually to the entire country. For the successful control and prevention of rabies in Indonesia, there is an urgent need to invest in strengthening health-care services and rabies surveillance activities.

## Conclusion

The findings of the current study show that most of the participants had sufficient knowledge and appropriate practices regarding rabies. However, there are some gaps in knowledge and practices among the participants, especially on rabies vaccination survey, doctors visiting the affected areas (that create awareness related to rabies in terms of treatment and prevention), and using any medication at home after a dog bite. With the help of vaccination programs, community forums, and media distribution of information, we have recognized a critical need to raise awareness of rabies. Such interventions may be effective in improving human attitudes to seeking medical care before and after a dog bite. Such study results may help to improve rabies policies and targeted management strategies in Indonesia to avoid rabies-related deaths.

## Data Availability

Supplementary data can be available from the corresponding author upon a reasonable request.

## Authors’ Contributions

SR and FAR: Conceptualization. SR, FAR, and MHE: Data curation. SR and AS: Formal analysis. MHE and FAR: Funding acquisition. SR, FAR, and MHE: Investigation. SR and FAR: Methodology. SR, FAR, and MHE: Project administration. SR, FAR, MHE, AR, and AS: Resources. FAR: Software. MHE and AS: Supervision. SR, FAR, MHE, and AS: Validation. FAR, MHE, and AS: Visualization. SR, AR and AS: Writing original draft. SR, MHE, AR and AS: Writing – review and editing. SR and AS: Map of study. All authors read and approved the final manuscript.
